# Inverse Lichen Planus Associated With Nail Dystrophy

**DOI:** 10.7759/cureus.49823

**Published:** 2023-12-02

**Authors:** Yasser Alrefaie, Ali Alraddadi, Yazeed Alhathal, Hanaa Bamefleh

**Affiliations:** 1 Dermatology Department, King Saud Medical City, Riyadh, SAU; 2 Dermatology Department, King Abdulaziz Medical City, Riyadh, SAU; 3 Dermatology Department, King Salman Hospital, Riyadh, SAU; 4 Pathology Department, King Saud Bin Abdulaziz University for Health Sciences, Riyadh, SAU; 5 Pathology Department, King Abdulaziz Medical City, Riyadh, SAU

**Keywords:** dystrophic nail, lichen planus, dorsal pterygium, nail lichen planus, inverse lichen planus

## Abstract

Lichen planus is a chronic inflammatory disorder affecting skin and mucosal surfaces. There are multiple variants of lichen planus described in the literature. We report a case of inverse lichen planus in a healthy 50-year-old male who presented to our dermatology clinic with multiple violaceous to hyperpigmented patches affecting both axillae and groin for three months. A skin biopsy confirmed the diagnosis of lichen planus. The patient subsequently developed nail dystrophy affecting his fingernails consistent with nail lichen planus. Early recognition and treatment of nail lichen planus is important to prevent irreversible scarring.

## Introduction

Lichen planus is an idiopathic inflammatory skin disorder that typically presents with pruritic, polygonal, violaceous papules and plaques on flexor surfaces of forearms, wrists, genitalia and oral mucosa [1]. There are more than 20 variants of lichen planus including oral, nail, annular, linear, hypertrophic, actinic, inverse, bullous, eruptive, lichen planus-lupus erythematosus overlap syndrome, lichen planopilaris, lichen planus pigmentosus [2].

Inverse lichen planus is a rare variant of lichen planus that presents with pink to violaceous patches and plaques affecting axillae, groin and inframammary areas [3]. Hyperpigmentation is mostly present, which can overlap with lichen planus pigmentosus [4].

Nail lichen planus has been reported in up to 15% of patients with lichen planus [5]. It can present findings affecting both the nail matrix and the nail bed. These findings include longitudinal ridging and splitting, trachyonychia, onycholysis and dorsal pterygium [6].

## Case presentation

A 50-year-old male presented to the clinic with a three-month history of itchy rash over both axillae and groin. No other skin, mucosal, scalp, or nail involvement at initial presentation. He had no past medical history and no systemic symptoms. Physical examination revealed multiple violaceous to hyperpigmented patches, with no secondary changes.

**Figure 1:**
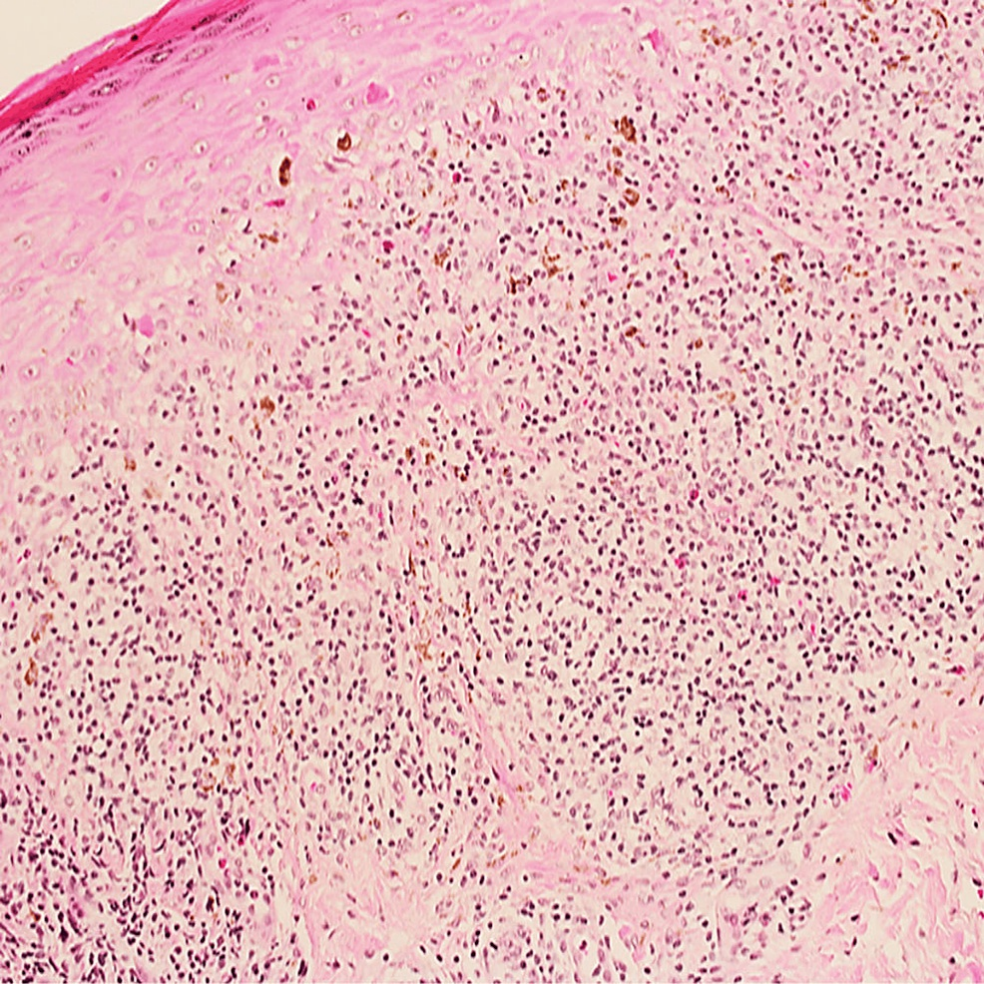
Histopathology findings from left axilla Band-like lymphocytic infiltrates at the dermo-epidermal junction with necrotic keratinocytes are seen at the lower epidermis.

A punch biopsy was taken from the left axilla revealed band-like lymphocytic infiltrates at the dermo-epidermal junction with hyperkeratosis, hypergranulosis and necrotic keratinocytes (Civatte bodies) visible at the lower epidermis (Figure 1).

**Figure 2:**
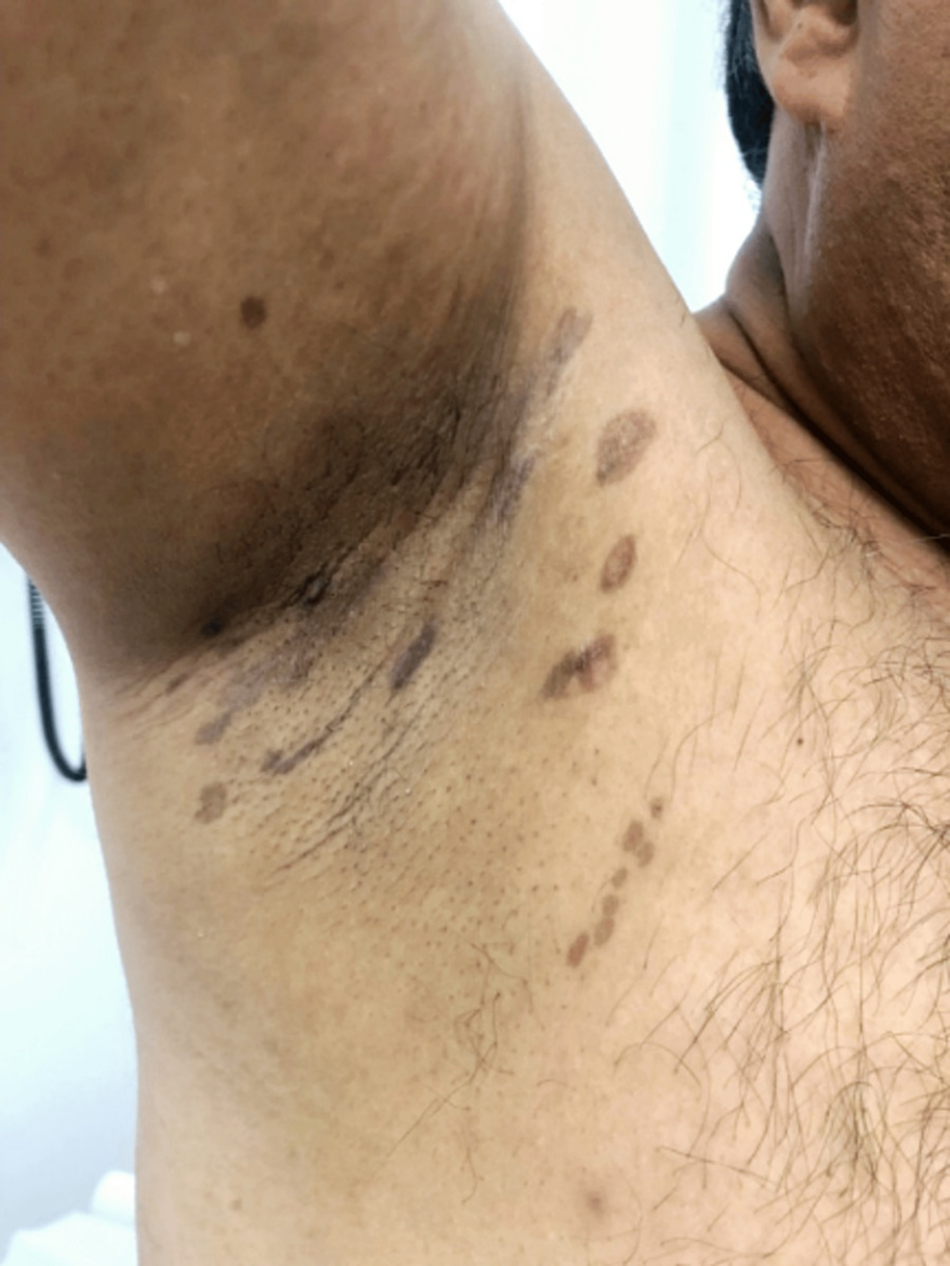
Right axilla Post-inflammatory hyperpigmentation is seen over the right axilla on follow-up.

**Figure 3:**
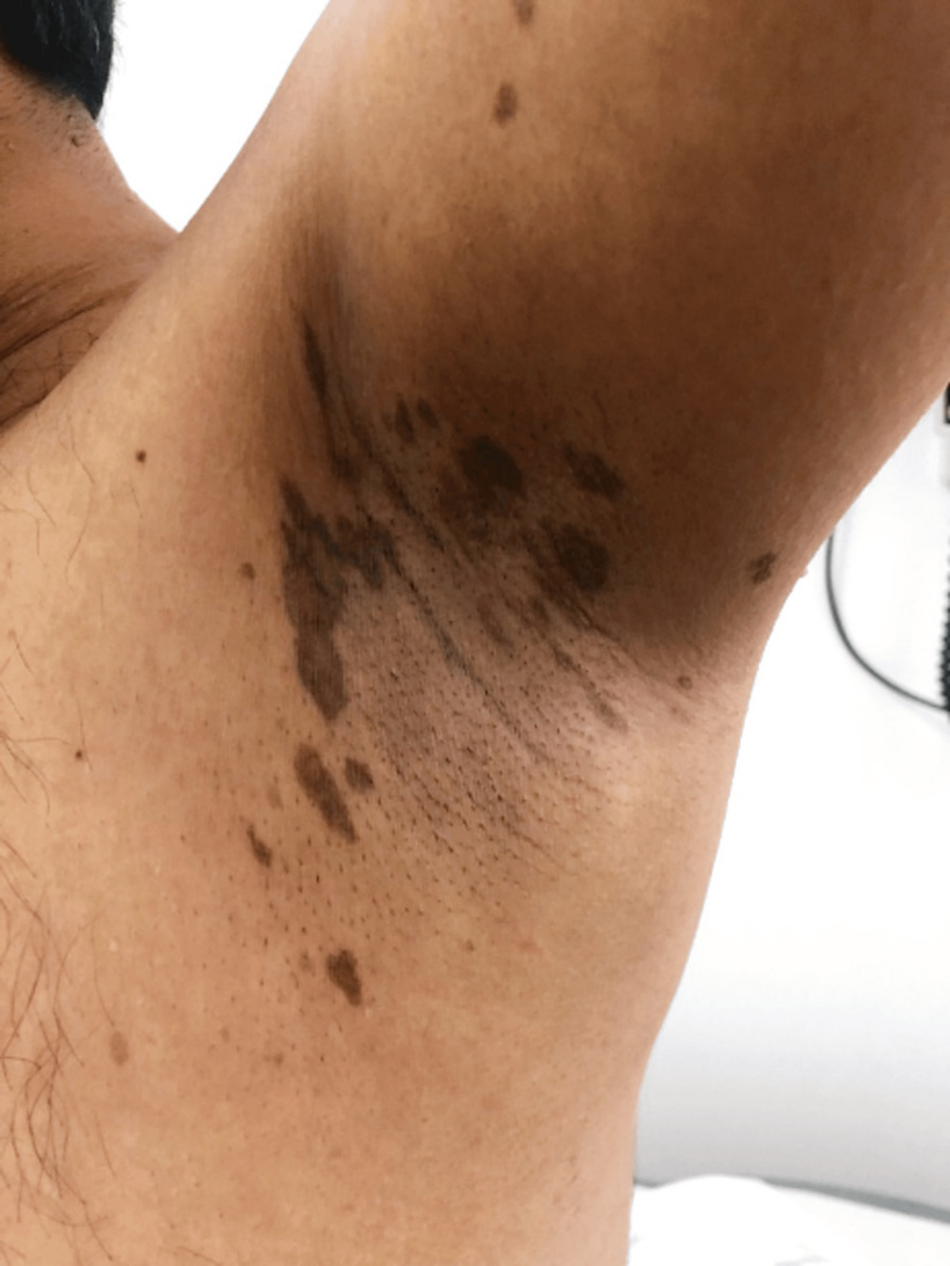
Left axilla Post-inflammatory hyperpigmentation is seen over the left axilla on follow-up.

**Figure 4:**
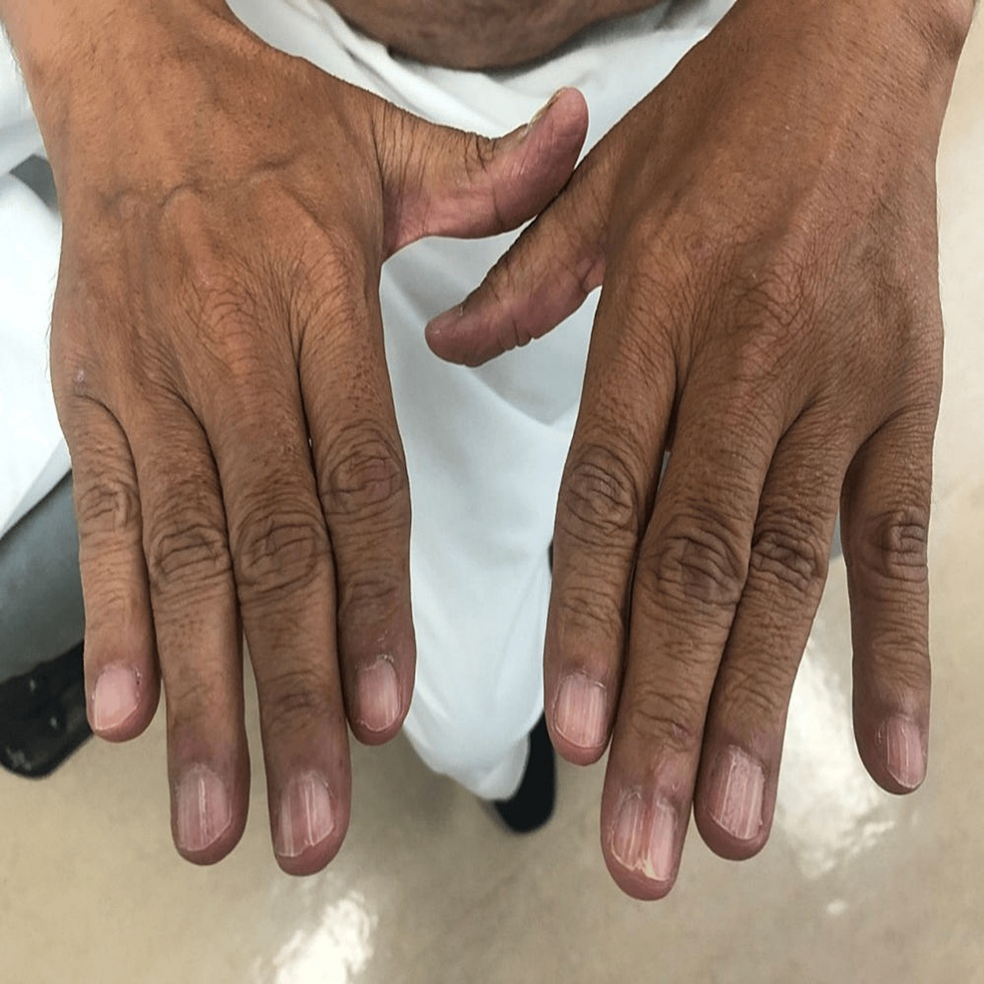
Nail lichen planus in fingernails Longitudinal ridging, splitting and trachyonychia affecting fingernails. Dorsal pterygium can be seen in the left middle fingernail.

**Figure 5:**
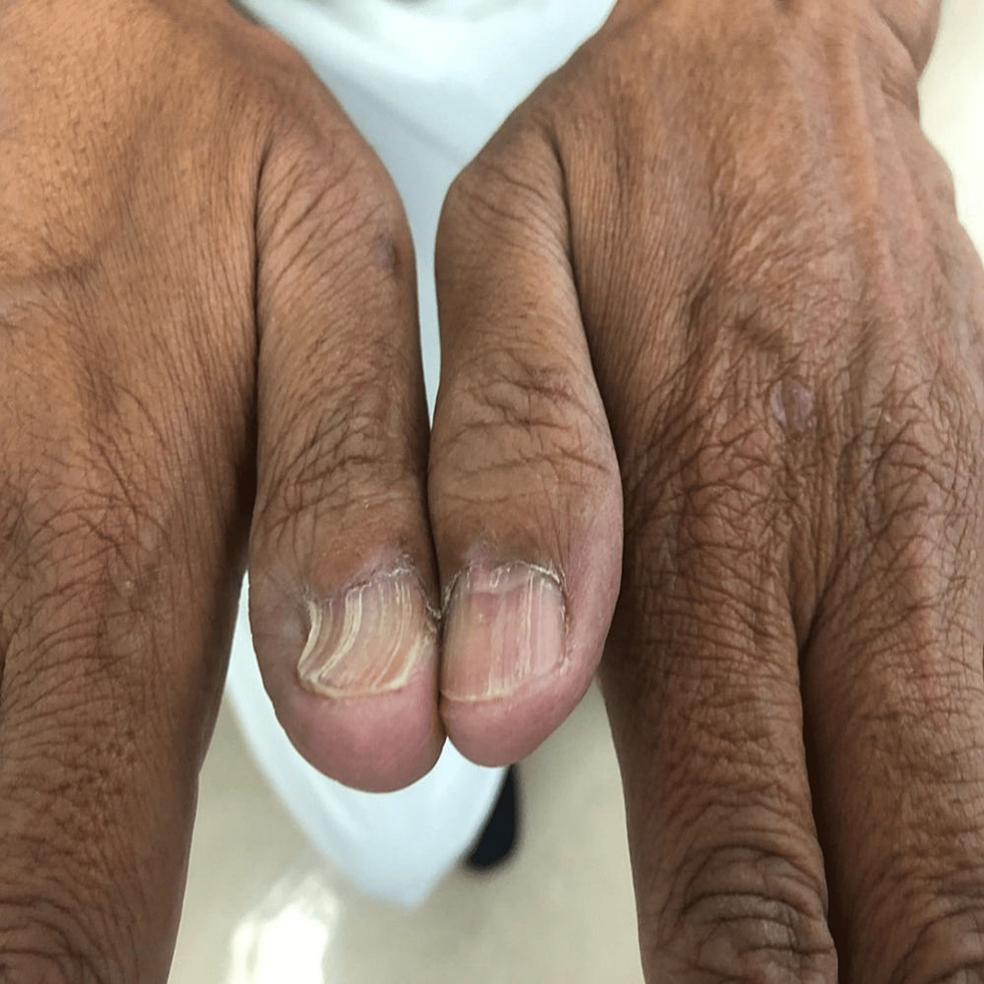
Nail lichen planus in thumbnails Longitudinal ridging in both thumbnails.

The patient was started on tacrolimus 0.1% ointment twice daily for two months which resulted in improvement of the lesions with only post-inflammatory hyperpigmentation (Figures 2-3). One year after the initial presentation, the patient developed dystrophic nail changes with longitudinal ridging, splitting, trachyonychia and dorsal pterygium (Figures 4-5). The patient was offered systemic immunosuppressant treatment, however, he refused treatment.

## Discussion

Inverse lichen planus is a rare variant of lichen planus with unusual morphological presentation affecting the axillae, inguinal folds and inframammary areas [3]. Sites of inverse lichen planus and its occluded nature can make the presentation different from the classic lichen planus [2]. Lesions can be without scales and more erythematous which can make the clinical diagnosis challenging [7].

There can be an overlap between inverse lichen planus and lichen planus pigmentosus. Lichen planus pigmentosus typically presents as hyperpigmented macules and patches affecting sun-exposed sites [4]. In our patient, the initial presentation with violaceous patches and localized distribution to intertriginous areas favour the diagnosis of inverse lichen planus.

The literature review showed only a few reported cases of Inverse lichen planus [8-12]. None of the reported cases was associated with nail dystrophy. This is to the best of our knowledge the first reported case of inverse lichen planus associated with nail dystrophy.

Nail lichen planus is an inflammatory process that affects the nail unit [13]. Treatment of nail involvement can be challenging as the use of topical treatment is not expected to have a major effect due to poor absorption into the nail matrix and bed [13]. Multiple treatment modalities were described in the literature including intralesional and intramuscular triamcinolone acetonide, acitretin, and immunosuppressive agents. Early diagnosis and treatment of nail lichen planus is necessary to prevent permanent scarring [14].

## Conclusions

Lichen planus is a chronic inflammatory papulosquamous disorder. Inverse lichen planus is a rare variant affecting intertriginous areas. Treatment of nail lichen planus can be challenging and early intervention should be considered to prevent irreversible nail damage.
